# Protein crystallization with microseed matrix screening: application to human germline antibody Fabs

**DOI:** 10.1107/S2053230X14012552

**Published:** 2014-07-23

**Authors:** Galina Obmolova, Thomas J. Malia, Alexey Teplyakov, Raymond W. Sweet, Gary L. Gilliland

**Affiliations:** a Janssen Research and Development LLC, 1400 McKean Road, Spring House, PA 19477, USA

**Keywords:** Fabs, crystallization, microseed matrix screening, seeding, optimization, automation

## Abstract

The crystallization of 16 human antibody Fab fragments constructed from all pairs of four different heavy chains and four different light chains was enabled by employing microseed matrix screening (MMS). In initial screening, diffraction-quality crystals were obtained for only three Fabs, while many Fabs produced hits that required optimization. Application of MMS, using the initial screens and/or refinement screens, resulted in diffraction-quality crystals of these Fabs. Five Fabs that failed to give hits in the initial screen were crystallized by cross-seeding MMS followed by MMS optimization. The crystallization protocols and strategies that resulted in structure determination of all 16 Fabs are presented. These results illustrate the power of MMS and provide a basis for developing future strategies for macromolecular crystallization.

## Abstract 2

The power of microseed matrix screening is demonstrated in the crystallization of a panel of antibody Fab fragments.

## 1. Introduction^1^   

Crystallization of macromolecules uses a set of experimental techniques aimed at producing crystals suitable for structure determination. Advances in molecular biology and X-ray data-collection methods are simplifying the task by providing pure proteins in large quantities and by utilizing smaller crystals, respectively. Crystallization methods have also improved over the years through the introduction of standard screens (Carter & Carter, 1979 ▶; Jancarik & Kim, 1991 ▶; Cudney *et al.*, 1994 ▶) and the use of robotics, which allows the screening of a large number of crystallization conditions in a miniaturized format, reducing the amount of protein needed (Stevens, 2000 ▶; Snook *et al.*, 2000 ▶; Weselak *et al.*, 2003 ▶; Rupp, 2003 ▶).

Another major development was the application of various microseeding techniques (Stura & Wilson, 1990 ▶; Stura, 1999 ▶; Bergfors, 2003 ▶). Seeding exploits the hypothesis that the optimal conditions needed for crystal nucleation and for crystal growth can be quite different (Kam *et al.*, 1978 ▶). Traditionally, two general approaches, microseeding and macroseeding, have been used to produce crystals of the macromolecule of interest (Bergfors, 2003 ▶). In some cases, cross-seeding, *i.e.* seeding with the crystals of a protein variant or a homologous protein, has proven successful (Stura & Wilson, 1991 ▶; Walter *et al.*, 2008 ▶). This approach can be used for related proteins that may include complexes with various ligands, heavy-atom derivatives and structurally similar proteins such as the Fab fragments of antibodies.

The seeding technique has been extended by the microseed matrix screening (MMS) approach in which seeds are systematically transferred into new conditions to promote crystal growth (Ireton & Stoddard, 2004 ▶). The screening process has been revolutionized by combining MMS with automation (D’Arcy *et al.*, 2007 ▶). Several successful applications of MMS have recently been reported (Obmolova *et al.*, 2010 ▶; Abskharon *et al.*, 2011 ▶; Malia *et al.*, 2011 ▶).

In this paper, we detail our experience with MMS in crystallization of antibody Fab fragments. With the number of Fab structures deposited in the Protein Data Bank (PDB) exceeding 1000, their crystallization is often considered straightforward, even though the PDB does not provide any information on how much effort was put into the crystallization of a particular Fab and how many attempts were unsuccessful. Two factors may be considered as favoring Fab crystallization: the availability of the protein owing to high expression levels and the flexibility of the two-domain structure that allows Fab molecules to adjust to crystal lattice constraints. With this in mind, we find this class of proteins particularly suitable for the evaluation of strengths and weaknesses of MMS.

**Figure 1:**
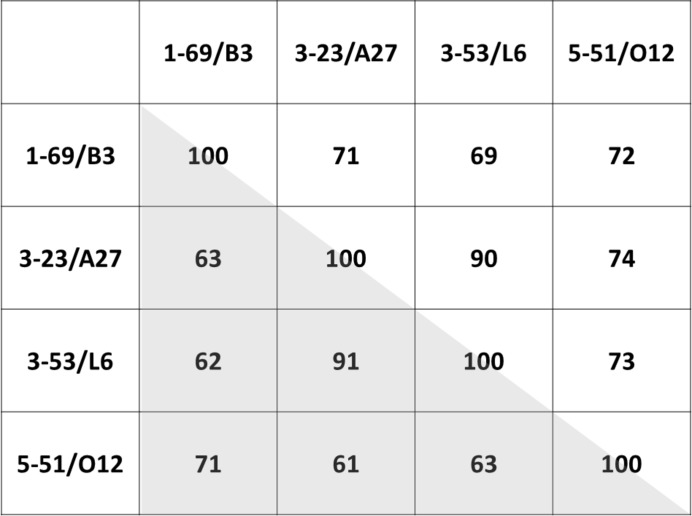
Sequence identity of VL and VH. Values for VL are in the upper right triangle; values for VH are in the lower left (shaded) triangle. For instance, the sequence identity between 3-23 and 5-51 is 61% and that between B3 and L6 is 69%.

The antibody Fabs for our study were produced as part of an effort to structurally and functionally characterize the human germline antibodies utilized in a phage-display combinatorial library at Janssen (Shi *et al.*, 2010 ▶). The variable domains of the Fabs were constructed from the germline sequences using a fixed H-CDR3 sequence. The constant domains of the Fabs were of the human IgG1/κ isotype. Four germlines were selected for each of the heavy and light chains, and all 16 combinations were expressed. The Fabs cover a wide range of sequences with 61–91% identity in the variable domain (Fig. 1 ▶).

All 16 Fabs were crystallized and their structures have been determined. We present the details of the crystallization process and discuss the application of MMS using both self-seeding and cross-seeding approaches.

## 2. Materials and methods   

### 2.1. Proteins   

The human germlines for the light chain were IGKV4-1, IGKV3-20, IGKV3-11 and IGKV1-39 according to the IMGT notation (Lefranc, 2001 ▶), which correspond to B3, A27, L6 and O12, respectively, in the V BASE nomenclature (Tomlinson *et al.*, 1998 ▶). The heavy-chain germlines were IGHV1-69, IGHV3-23, IGHV3-53 and IGHV5-51, which correspond to 1-69, 3-23, 3-53 and 5-51. The third complementarity-determining region (CDR) of the heavy chain, which normally results from recombination of the variable (V), diversity (D) and junction (J) genes, had the same amino-acid sequence in all Fabs, namely the sequence of the anti-CCL2 antibody CNTO 888 (Obmolova *et al.*, 2012 ▶): YDGIYGELDF. A 6×His tag was attached at the C-terminus of the heavy chains to facilitate purification.

The Fab fragments were expressed in HEK293E cells and purified using affinity and size-exclusion chromatography as described by Zhao *et al.* (2009 ▶). The proteins were dialyzed into 20 m*M* Tris buffer pH 7.4 with 50 m*M* NaCl and concentrated to 15 mg ml^−1^ using an Amicon Ultra 10 kDa MWCO device.

### 2.2. Crystallization   

Crystallization was carried out using the vapor-diffusion method at 20°C. Automated crystallization screening was performed with an Oryx4 (Douglas Instruments) or a Mosquito (TTP Labtech) crystallization robot in a sitting-drop format using Corning 3550 plates. Seed stocks were prepared by mechanical homogenization of crystals using the Seed Bead Kit (Hampton Research) as described by Luft & DeTitta (1999 ▶). Crystals were suspended in 50 µl reservoir solution containing the seed bead followed by stirring for 3 min using a laboratory vortex. The seed-stock solutions were stored at −20°C.

Crystallization droplets were composed of 0.2 µl protein solution and 0.2 µl reservoir solution. When seeding was applied, the droplets consisted of 0.2 µl protein solution, 0.15 µl reservoir solution and 0.05 µl seed solution. The droplets were equilibrated against 80 µl reservoir solution, typically for 3–5 d. Initial screening for crystallization conditions was performed with the 96-well Crystal Screen HT (Hampton Research) and an in-house 192-well screen optimized for Fab crystallization. The in-house screen contains polyethylene glycol (PEG) or salt conditions in a wide range of pH and PEG plus salt conditions similar to the PEG/Ion screen from Hampton Research but with two PEG concentrations rather than one. Unless otherwise noted, MMS was performed in the same two screens. Refinement screens were designed around the selected conditions by varying the precipitant concentration and the pH and by using additives such as 2-methyl-2,4-pentanediol (MPD), PEG 400 and dioxane.

### 2.3. X-ray analysis   

Crystals were cryocooled and assessed using a Rigaku MicroMax-007 HF microfocus X-ray generator equipped with a Saturn 944 CCD detector. Those with diffraction suitable for structure determination were considered to be ‘X-ray quality’ (XQ) crystals. All data were collected on the rotating-anode generator. Crystal data are given in Table 1 ▶.

## 3. Results   

**Figure 2:**
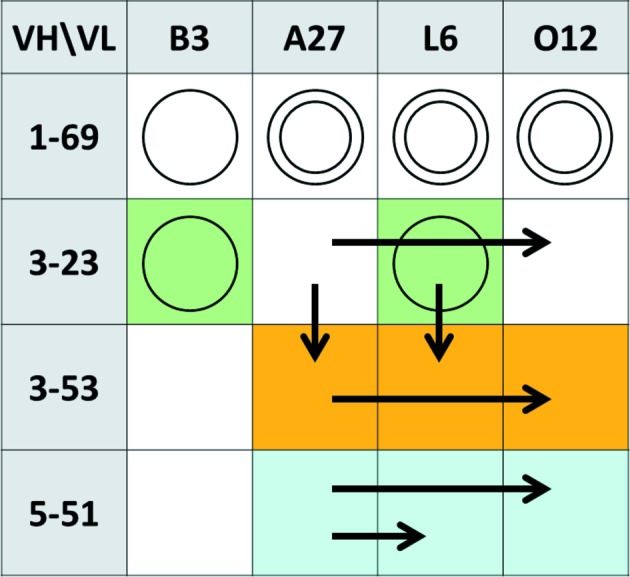
Summary of crystallization experiments. Single circles mark Fabs that gave XQ crystals in the initial screening. Double circles mark Fabs that only produced hits with double protein concentration. Cells of the same color indicate identical crystal forms. Successful cross-seeding is shown by arrows, where the tail marks the source of the seeds and the arrowhead marks the Fab that was crystallized.

All 16 Fabs were crystallized and their structures were solved. Of the 16, only three Fabs (Fig. 2 ▶) were crystallized directly from the initial screen and required no improvement. Five Fabs produced some hits in the initial screening that were used for self-seeding MMS. Eight Fabs gave no hits under the initial conditions. Five of them were successfully crystallized by cross-seeding MMS. The remaining three Fabs, all with the 1-69 heavy chain, failed in the cross-seeding experiments but produced hits in the initial screens when the protein concentration was increased twofold to 30 mg ml^−1^. XQ crystals were then obtained using self-seeding MMS.

### 3.1. Initial screening   

Initial screening using the 96-well Crystal Screen HT and the in-house 192-well screen generated hits for eight of the Fabs. Three of these, Fabs 3-23/B3, 3-23/L6 and 1-69/B3, gave XQ crystals in these experiments. Interestingly, crystals of 3-23/B3 and 3-23/L6 that share the same VH were obtained under similar conditions from 2 *M* AmSO4 and are isomorphous (Table 1 ▶). In this crystal form, L-CDR1, which has a six-residue insertion in B3 with respect to L6, is not involved in crystal contacts.

### 3.2. Self-seeding MMS   

Five Fabs gave no XQ crystals in the initial screens but produced some hits that could be optimized by MMS using either a random or a refinement screen. These Fabs included 3-23/A27, 3-53/B3, 3-53/L6, 5-51/A27 and 5-51/B3. For two of them, 3-23/A27 and 5-51/A27, XQ crystals were obtained after a second round of MMS using a tenfold-diluted seed stock solution. Details for each of the five Fabs are presented below.

#### 3.2.1. Fab 3-23/A27   

**Figure 3:**
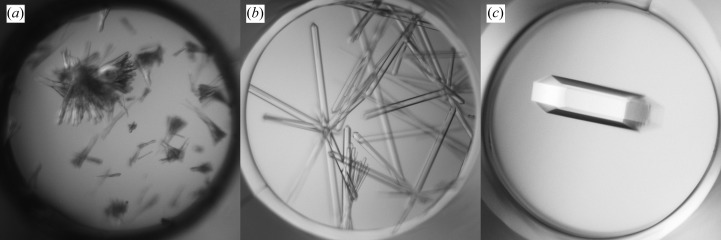
Crystallization of Fab 3-23/A27. (*a*) Initial screening hit in 18% PEG 3350, 0.2 *M* NaAct, 0.1 *M* MES pH 6.5. (*b*) Crystals obtained by MMS in 18% PEG 3350, 1.0 *M* LiCl, 0.1 *M* MES pH 6.5. (*c*) The same as (*b*) but after diluting the seeds tenfold. The images were produced by Crystal Farm (Bruker AXS) or Rock Imager (Formulatrix). The diameter of the well is 1 mm.

The initial screening produced needle-like crystals in solutions containing PEG with salt at various pH values (Fig. 3 ▶ *a*). A seed stock was prepared by mixing crystals from conditions consisting of 18% PEG 3350, 0.1 *M* MES pH 6.5 and either 0.2 *M* NaAct or 0.2 *M* NaFmt. The first round of MMS in the initial screens produced XQ crystals in 18% PEG 3350, 0.1 *M* MES pH 6.5 and either 1.0 *M* LiCl or 0.2 *M* MgCl_2_ (Fig. 3 ▶ *b*). Larger crystals were obtained when the seed stock was diluted tenfold (Fig. 3 ▶ *c*).

#### 3.2.2. Fab 3-53/B3   

The initial screening produced crystal clusters in solutions containing PEG with salt at pH 6.5–7.5. A seed stock was prepared by mixing crystals from three conditions consisting of 18% PEG 3350, 0.1 *M* MES pH 6.5 and 0.2 *M* salt (LiCl, NaAct or NaFmt). MMS optimization with a refinement screen yielded XQ crystals in many wells. The crystal used for X-ray analysis was obtained from 16% PEG 3350, 0.2 *M* NaFmt, 5% MPD, 0.1 *M* MES pH 6.5.

#### 3.2.3. Fab 3-53/L6   

**Figure 4:**
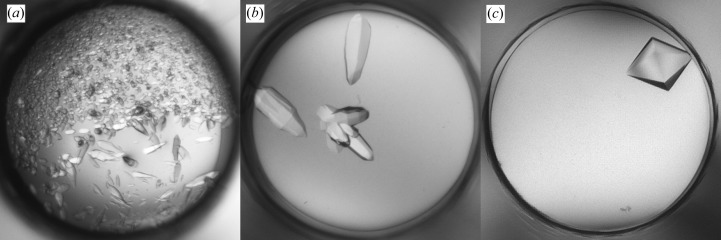
Crystallization of Fab 3-53/L6. (*a*) Initial screening hit in 30% PEG 4000, 0.2 *M* AmSO4. (*b*) Crystals in 10% PEG 4000, 0.2 *M* AmSO4 obtained by self-seeding MMS in the refinement screen. (*c*) Crystal in 25% PEG 3350, 0.2 *M* Li_2_SO_4_, 0.1 *M* NaAct pH 4.5 obtained by cross-seeding MMS in the initial screen.

The initial screening produced a shower of crystals in 30% PEG 4000, 0.2 *M* AmSO4 (Fig. 4 ▶ *a*) and these crystals were used to make seeds. Microseeding was performed in the refinement screen with 0.2 *M* AmSO4 in all conditions, whereas the PEG concentration and pH were varied. Multiple hits were observed including XQ crystals in 10% PEG 4000, 0.2 *M* AmSO4 which diffracted to 2.8 Å resolution (Fig. 4 ▶ *b*). Alternatively, MMS was performed in the initial screen using seeds from Fab 3-23/L6 obtained in 2.0 *M* AmSO4, 0.1 *M* NaAct pH 4.5. This approach produced XQ crystals in various conditions containing 18–25% PEG 3350, 0.2 *M* salt (NaAct or Li_2_SO_4_) and 0.1 *M* buffer (NaAct pH 4.5 or Tris pH 8.5) (Fig. 4 ▶ *c*). Both approaches resulted in the same crystal form, although the shape of the crystals was different and the latter crystals diffracted much better to 2.3 Å resolution.

#### 3.2.4. Fab 5-51/A27   

**Figure 5:**
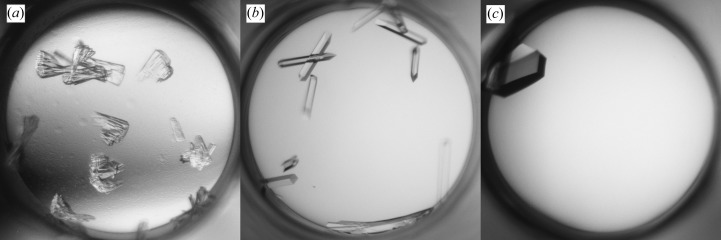
Crystallization of Fab 5-51/A27. (*a*) Initial screening hit in 2.0 *M* AmSO4, 5% PEG 400, 0.1 *M* Tris pH 8.5. (*b*) Crystals in 2.0 *M* AmSO4, 0.1 *M* CHES pH 9.5 obtained by MMS. (*c*) Crystal in 1.0 *M* AmSO4, 0.1 *M* CHES pH 9.5 from refinement screening with diluted seeds.

The initial screening produced large crystal clusters in solutions containing AmSO4 at pH 8.5–9.5 (Fig. 5 ▶ *a*). Crystals from 2.0 *M* AmSO4, 5% PEG 400, 0.1 *M* Tris pH 8.5 were selected to prepare a seed stock. MMS in the initial screen gave XQ crystals under several conditions, the best being from 2.0 *M* AmSO4, 0.1 *M* CHES pH 9.5 (Fig. 5 ▶ *b*). They were further improved using the seed stock diluted tenfold. This yielded crystals in 1.0 *M* AmSO4, 0.1 *M* CHES pH 9.5 that diffracted to 1.6 Å resolution (Fig. 5 ▶ *c*).

#### 3.2.5. Fab 5-51/B3   

**Figure 6:**
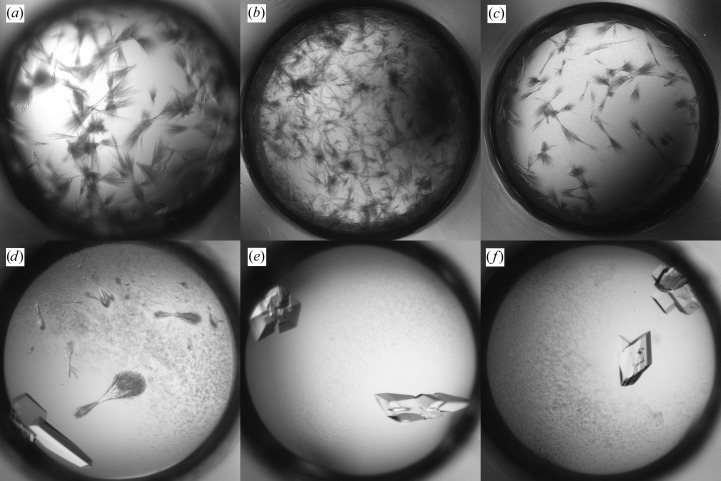
Crystallization of Fab 5-51/B3. (*a*, *b*, *c*) Hits from the initial screening; (*d*, *e*, *f*) crystals obtained with MMS. (*a*) 25% PEG 3350, 0.2 *M* Li_2_SO_4_, 0.1 *M* Tris pH 8.5. (*b*) 18% PEG 3350, 0.2 *M* AmAct, 0.1 *M* Tris pH 8.5. (*c*) 18% PEG 8000, 0.1 *M* MES pH 6.5. (*d*) 24% PEG 3350, 0.2 *M* AmAct, 0.1 *M* Tris pH 8.5. (*e*) 25% PEG 3350, 0.2 *M* NH_4_Cl, 0.1 *M* Tris pH 8.5. (*f*) 18% PEG 3350, 0.2 *M* NH_4_Cl, 0.1 *M* HEPES pH 7.5.

The initial screening produced needle-like crystals in various PEG-containing solutions at pH 6.5–8.5. The seed stock was prepared by mixing crystals from the conditions shown in Figs. 6 ▶(*a*), 6 ▶(*b*) and 6 ▶(*c*). MMS was performed in the same screen and yielded XQ crystals in similar conditions (Figs. 6 ▶ *d*, 6 ▶ *e* and 6 ▶ *f*). The crystal shown in Fig. 6 ▶(*d*) was used to determine the structure at 2.0 Å resolution.

### 3.3. Cross-seeding MMS   

The initial screening for eight of the Fabs produced no hits at all. Of these, five Fabs, 3-23/O12, 3-53/A27, 3-53/O12, 5-51/L6 and 5-51/O12, produced hits in cross-seeding experiments. The cross-seeding MMS approaches described below vary in the source of seeds from a single Fab to seed mixtures from two or more Fabs. For all but one of the Fabs, 5-51/L6, the crystals from the cross-seeding MMS were used for the secondary MMS experiments. In some cases multiple rounds of optimization MMS were required.

#### 3.3.1. Fab 3-23/O12   

**Figure 7:**
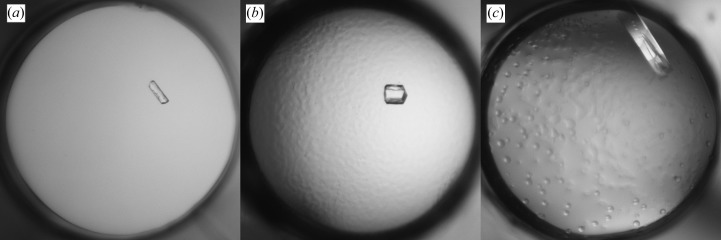
Crystallization of Fab 3-23/O12. (*a*) Crystal in 18% PEG 3350, 0.2 *M* AmTrt obtained by cross-seeding MMS. (*b*) Crystal in 24% PEG 3350, 1.0 *M* LiCl, 0.1 *M* NaAct pH 4.5 obtained from the second round of MMS with self-seeds. (*c*) Crystal in 20% PEG 3350, 0.2 *M* LiCtr obtained from the third round of MMS with self-seeds.

MMS with the Fab 3-23/A27 seeds described above produced one small crystal in 18% PEG 3350, 0.2 *M* AmTrt (Fig. 7 ▶ *a*), which was used as a source of seeds in a second round of MMS. A new hit was obtained in 24% PEG 3350, 1.0 *M* LiCl, 0.1 *M* NaAct pH 4.5 (Fig. 7 ▶ *b*). This crystal was used to make seeds for a third round of MMS. This was performed in the PEG/Ion screen and yielded an XQ crystal in 20% PEG 3350, 0.2 *M* LiCtr (Fig. 7 ▶ *c*).

#### 3.3.2. Fab 3-53/A27   

**Figure 8:**
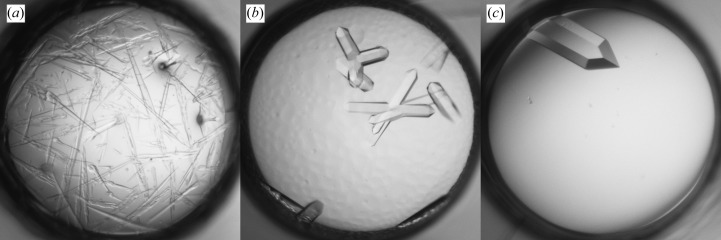
Crystallization of Fab 3-53/A27. (*a*) Crystals from MMS with Fab 3-23/A27 seeds in 30% PEG 4000, 0.2 *M* AmSO4. (*b*) Crystals from MMS with self-seeds in 2.0 *M* AmSO4, 0.1 *M* NaAct pH 4.5. (*c*) Crystals from refinement screening with self-seeds in 19% PEG 4000, 0.2 *M* AmSO4, 0.1 *M* NaAct pH 4.5.

In the cross-seeding MMS experiments for this Fab, two sets of seeds were used: one produced from crystals of Fab 3-23/A27 (the same light chain) and the other from Fab 3-53/B3 (the same heavy chain). The seed stocks contained mixtures of different hits from similar conditions (18% PEG 3350, 0.2 *M* salt, 0.1 *M* MES pH 6.5) as described above. MMS with the Fab 3-53/B3 seeds gave no hits. However, MMS with the Fab 3-23/A27 seeds gave one hit in 30% PEG 4000, 0.2 *M* AmSO4 (Fig. 8 ▶ *a*). These conditions are quite different from the seed-stock solution, which contained 18% PEG 3350, 0.2 *M* NaAct/NaFmt and 0.1 *M* MES pH 6.5. The needle-like crystals of Fab 3-53/A27 were converted into new seeds and MMS was repeated in the same screens. In this round, XQ crystals were observed in 24 out of 288 conditions (Fig. 8 ▶ *b*). The same seeds were applied in the refinement screening around some of the new conditions, which yielded a larger and better diffracting crystal that was used for structure determination (Fig. 8 ▶ *c*).

#### 3.3.3. Fab 3-53/O12   

A seed mixture for MMS was prepared by combining the seeds from the crystals of four different Fabs obtained under the following conditions: 18% PEG 4000, 0.2 *M* AmSO4, 0.1 *M* NaAct pH 4.5 (3-53/A27), 18% PEG 3350, 0.2 *M* NaFmt, 0.1 *M* MES pH 6.5 (3-53/B3), 18% PEG 3350, 1.0 *M* LiCl, 0.1 *M* MES pH 6.5 (3-23/A27) and 18% PEG 3350, 0.2 *M* AmSO4, 0.1 *M* Tris pH 8.5 (5-51/B3). MMS with this seed mixture produced a number of hits, two of which were selected to make new seeds: 30% PEG 8000, 0.2 *M* AmSO4 and 30% PEG 4000, 0.2 *M* AmSO4. Refinement screening with the self-seed mixture yielded XQ crystals in 16% PEG 3350, 0.2 *M* AmSO4, 5% dioxane that appeared to be isomorphous to the Fab 3-53/A27 crystals.

#### 3.3.4. Fab 5-51/L6   

Two sets of seeds were used for the MMS. The first was prepared from crystals of a Fab with the same light chain, 3-53/L6, that were grown in 10% PEG 4000, 0.2 *M* AmSO4, 0.1 *M* NaCtr pH 3.4. MMS with these seeds failed to produce hits. However, a seed stock made from crystals of Fab 5-51/A27, which shares the same VH (grown in 1.2 *M* AmSO4, 0.1 *M* CHES pH 9.5), produced several XQ crystals. The crystal obtained in 25% PEG 3350, 0.2 *M* MgCl_2_, 0.1 *M* Tris pH 8.5 was used for structure determination and appeared to be isomorphous to the seeding crystals of Fab 5-51/A27.

#### 3.3.5. Fab 5-51/O12   

**Figure 9:**
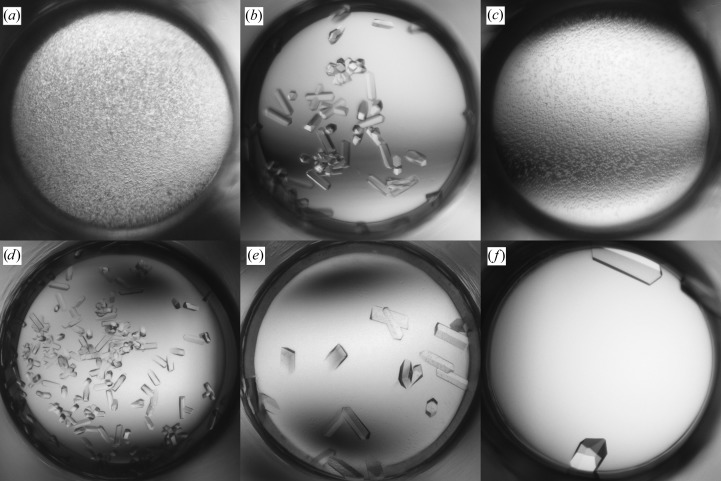
Crystallization of Fab 5-51/O12. (*a*)–(*e*) MMS results in 2.0 *M* AmSO4, 0.1 *M* CHES pH 9.5 with the following crystallization seeds: (*a*) AmSO4 + PEG mixture, (*b*) AmSO4 mixture, (*c*) AmSO4 mixture without Fab 5-51/A27, (*d*) only Fab 5-51/A27, (*e*) the same as (*d*) but with seeds diluted tenfold. (*f*) Crystals from refinement screening with the Fab 5-51/A27 seeds in 1.8 *M* AmSO4, 5% dioxane, 0.1 *M* CHES pH 9.5.

Seeds for cross-seeding MMS were prepared from the crystals of six other Fabs grown under various conditions. Three of them were obtained from AmSO4 solutions: 2.4 *M* AmSO4, 0.1 *M* NaAct pH 4.5 (3-23/L6), 2.0 *M* AmSO4, 0.1 *M* NaAct pH 4.5 (3-53/A27) and 2.0 *M* AmSO4, 5% PEG 400, 0.1 *M* Tris pH 8.5 (5-51/A27). The other three were obtained from PEG solutions: 16% PEG 3350, 0.2 *M* NaFmt, 0.1 *M* MES pH 6.5 (3-53/B3), 20% PEG 3350, 0.2 *M* AmAct, 0.1 *M* Tris pH 8.5 (5-51/B3) and 18% PEG 3350, 0.1 *M* LiCl, 0.1 *M* MES pH 6.5 (3-23/A27). MMS with a seed mixture containing crystals of the three Fabs obtained under the AmSO4 conditions produced well shaped crystals in 2.0 *M* AmSO4, 0.1 *M* CHES pH 9.5 (Fig. 9 ▶ *b*). The same type of crystals could be obtained when seeds were made from only one Fab, 5-51/A27 (Fig. 9 ▶ *d*). However, no hits were observed when the seed mixture contained just the two other ‘AmSO4’ Fabs, 3-23/L6 and 3-53/A27 (Fig. 9 ▶ *c*). This indicates that the Fab 5-51/A27 seeds, either alone or in the mixture, promoted crystal growth of Fab 5-51/O12. The seed mixture containing all six Fabs did not produce any hits in the same screen (Fig. 9 ▶ *a*), suggesting that the AmSO4 seeds may have dissolved in the presence of PEG.

Cross-seeding MMS was repeated with the Fab 5-51/A27 seeds that were diluted tenfold, leading to the production of fewer and larger crystals (Fig. 9 ▶ *e*). The same seeds were then used in refinement MMS with a screen designed around the conditions producing crystals. This yielded crystals that diffracted to 2.2 Å resolution (Fig. 9 ▶ *f*). These crystals appeared to be isomorphous to those of Fab 5-51/A27.

### 3.4. Screening with high protein concentration   

Fabs 1-69/A27, 1-69/L6 and 1-69/O12, all with the same heavy chain, did not give hits in the initial screen or in cross-seeding MMS. For these three Fabs, the protein concentration was increased to 30 mg ml^−1^ and the initial screens were rerun. In all three cases, hits were found which were then used to prepare seeds for further MMS experiments.

#### 3.4.1. Fab 1-69/A27   

**Figure 10:**
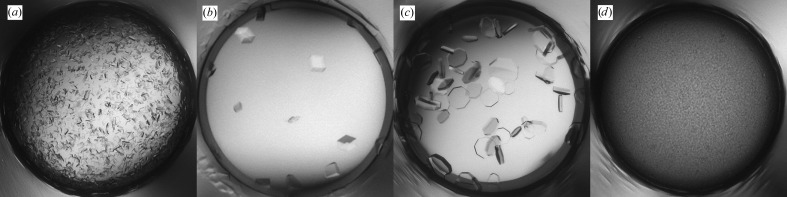
Crystallization of Fab 1-69/A27. (*a*) Hits from initial screening at 30 mg ml^−1^ in 2.0 *M* AmSO4, 5% MPD, 0.1 *M* MES pH 6.5. (*b*) Crystals in 2.0 *M* AmSO4, 5% MPD, 0.1 *M* Tris pH 8.5 obtained by MMS at 16 mg ml^−1^. (*c*, *d*) Results from refinement screening at 16 mg ml^−1^ with seeds from (*a*) and AmSO4 concentrations of 2.07 *M* (*c*) and 2.19 *M* (*d*).

The initial screening and cross-seeding MMS with the seed stocks obtained from Fabs 3-23/A27, 3-53/B3, 3-23/L6, 5-51/B3 and 5-51/A27 were unsuccessful. After raising the protein concentration from 15 to 30 mg ml^−1^ and rerunning the initial screens, a shower of microcrystals was observed after one month in 2.0 *M* AmSO4, 5% MPD, 0.1 *M* MES pH 6.5 (Fig. 10 ▶ *a*). Crystals from this experiment were used to make seeds for the next round of screening. MMS in the initial screen at 30 mg ml^−1^ protein concentration did not produce any hits, but when the protein concentration was reduced to 15 mg ml^−1^ small crystals appeared in 2.0 *M* AmSO4, 5% MPD, 0.1 *M* Tris pH 8.5 (Fig. 10 ▶ *b*). The same original seeds were also used in refinement screening with various buffers and AmSO4 concentrations. This resulted in either clear drops or precipitate. The refinement screening was repeated with a finer grid of AmSO4 concentration that was reduced from 0.26 to 0.12 *M*. This time XQ crystals were observed at 2.07 *M* AmSO4 (Fig. 10 ▶ *c*). Interestingly, at a lower (1.95 *M*) AmSO4 concentration the drop remained clear for at least three weeks, whereas heavy precipitate was observed in experiments at higher (2.19 *M*) AmSO4 concentrations (Fig. 10 ▶ *d*).

#### 3.4.2. Fab 1-69/L6   

**Figure 11:**
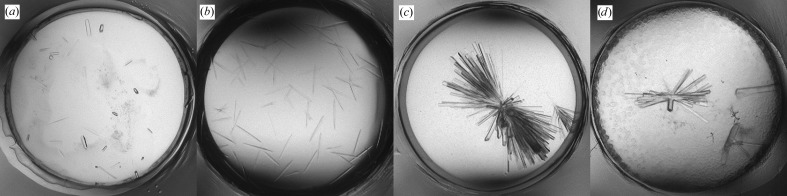
Crystallization of Fab 1-69/L6. (*a*) Small crystals in 2.8 *M* AmSO4, 5% MPD, 0.1 *M* Tris pH 8.5 obtained from initial screening at 30 mg ml^−1^. (*b*) Needles in 2.0 *M* AmSO4, 5% MPD, 0.1 *M* HEPES pH 7.5 obtained from refinement screening with seeds from (*a*) at 30 mg ml^−1^. (*c*) Crystals in 25% PEG 3350, 0.2 *M* LiCl, 0.1 *M* NaAct pH 4.5 obtained by MMS with seeds from (*b*). (*d*) Crystals in 25% PEG 3350, 0.2 *M* NaFmt, 0.1 *M* MES pH 6.5 obtained by MMS with seeds from (*b*).

The initial screening and cross-seeding with crystals of Fabs 3-23/L6, 5-51/A27 or 1-69/O12 was unsuccessful. A repeat of the initial screening at higher Fab concentration gave one hit in 2.8 *M* AmSO4, 5% MPD, 0.1 *M* Tris pH 8.5 (Fig. 11 ▶ *a*). These tiny crystals were converted into seeds that were used in optimization MMS, where the AmSO4 concentration was varied from 1.0 to 2.8 *M* and the pH was varied from 6.5 to 10.5. No significant improvement was observed; however, needle-like crystals were obtained in 2.0 *M* AmSO4, 5% MPD, 0.1 *M* HEPES pH 7.5 (Fig. 11 ▶ *b*) that became a source of a seed stock for the next round of screening. MMS with these new seeds using the initial screens produced XQ crystals under completely different conditions: 25% PEG 3350, 0.2 *M* LiCl, 0.1 *M* NaAct pH 4.5 (Fig. 11 ▶ *c*) and 25% PEG 3350, 0.2 *M* NaFmt, 0.1 *M* MES pH 6.5 (Fig. 11 ▶ *d*).

#### 3.4.3. Fab 1-69/O12   

MMS at the higher Fab concentration (30 mg ml^−1^) in the initial screens produced a shower of small crystals in 4.5 *M* NaFmt, 0.1 *M* MES pH 6.5. MMS with these seeds at a reduced protein concentration (15 mg ml^−1^) produced XQ crystals in the same conditions.

## 4. Discussion   

**Figure 12:**
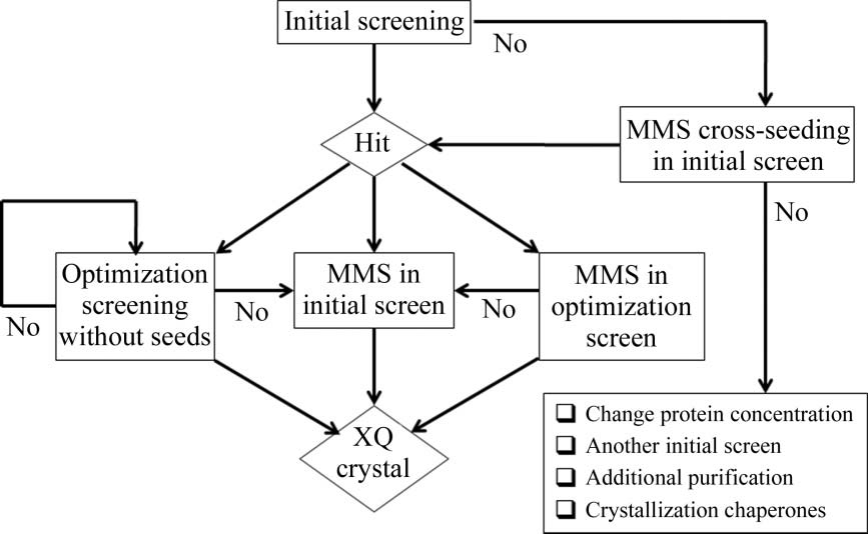
Workflow diagram of the crystallization strategies.

Protein crystallization involves two stages: nucleation and crystal growth. It is believed that the optimal conditions for these stages are different (García-Ruiz, 2003 ▶). Nucleation typically requires higher concentrations of the protein and precipitant. Seeding was devised as a means to decouple the two stages and promote crystal growth (Stura & Wilson, 1990 ▶). In a traditional approach to growing XQ crystals, the initial screening is followed by an optimization screening that is focused around the conditions that produced the initial hits (Fig. 12 ▶). This screening is usually performed over a narrow range of conditions using a fine but often multidimensional grid. However, it is not uncommon that the secondary screening shows no improvement in crystal quality and requires the optimization of a different hit (if available). In this unfortunate situation, protein and time will be wasted. It has been recognized that the introduction of microseeds into the optimization screening greatly increases the chances of success (Bergfors, 2003 ▶). The development and implementation of MMS has carried this even further, in the sense that the probability of success is further enhanced.

The MMS concept takes advantage of the fact that the conditions for crystal growth can differ from the conditions required for crystal nucleation. Thus, secondary screening with the addition of seeds is usually performed in the original screen, although screens with other sets of conditions can also be employed. This is then often followed by optimization MMS that employs the same optimization principles as described in the traditional crystallization approach but with the introduction of seeds. There are several advantages to this approach. Firstly, there is often no need to prepare a refinement screen for the hits in the initial screen, which takes time even when using a liquid-handling robot. Secondly, new and better hits may be obtained in conditions that are quite different from the initial hit. Thirdly, compared with optimization without seeds, the time to produce results is significantly reduced, typically to 2–3 d. The experiments described in this report highlight these advantages.

Eight Fabs out of 16 produced some hits in the first round of initial screening. While three of the Fabs produced crystals suitable for structure determination, the other five required optimization. In two cases, 3-53/B3 and 3-53/L6, microseeding was performed in a traditional way using a refinement screen designed around the crystallization conditions of the seeds. In four cases (3-23/A27, 3-53/L6, 5-51/A27 and 5-51/B3) MMS was employed in the initial screens covering a wide range of conditions. In both approaches XQ crystals were obtained, suggesting that either method is effective. The choice of the approach to pursue usually depends on the amount of protein, the number of hits and the time available. The screen used in MMS optimization may be relatively small and therefore requires less protein and fewer seeds. A sparse-matrix screen requires more protein but can save some time in screen preparation. MMS in the initial screen covers a wide range of conditions and therefore is a preferred starting point that may be followed by MMS optimization, if necessary.

An important advantage of MMS over traditional optimization screening is highlighted by the case of Fab 3-53/L6. Both approaches were applied in parallel after the initial round of screening. Seeding in the conditions around the initial hit produced XQ crystals, which have the same shape as the original crystals (Fig. 4 ▶). Alternatively, MMS in a random screen identified new conditions that produced crystals with a different morphology and much improved diffraction. These experiments indicate that optimization screening may improve the size of the crystals but not necessarily the shape. If the crystals are one-dimensional (needles) or two-dimensional (thin plates), MMS is worth trying in order to improve the crystal morphology.

In many cases MMS exceeded our expectations, but there was one instance where traditional optimization screening proved to be crucial for obtaining XQ crystals. The metastable zone of supersaturated crystallization solution is so narrow for Fab 1-69/A27 that the AmSO4 concentration has to be within 0.06 *M* of the optimal value. Obviously, in such cases the chance of hitting the target by random screening is too small and an MMS optimization with a fine grid is the way to proceed. Fortuitously, in this particular case the optimal concentration of 2.0 *M* was included in the random screen, which did produce some crystals.

Initial hits may be of a better or worse quality, but the real challenge is a lack of hits. This situation occurred in half of our experiments even though nearly 300 conditions were tried. Cross-seeding proved to be a very efficient technique to deal with the problem, but initially posed the challenge of which seeds to select. There are many suggestions in the literature ranging from protein crystals to polymer layers and mineral rocks (McPherson & Shlichta, 1988 ▶; Edwards *et al.*, 1994 ▶; Landau & Rosenbusch, 1996 ▶; Chayen *et al.*, 2001 ▶; Fermani *et al.*, 2001 ▶; D’Arcy *et al.*, 2003 ▶). Since our targets belong to one protein class (Fab fragments) and in many cases share a common light or heavy chain, it was natural to use available Fab crystals for seeding the others. In these experiments, mixtures of various seeds (‘polyseeds’) can save time and protein and can have a higher chance of success compared with a single attempt with ‘monoseeds’. It is probably preferable to mix seeds from similar conditions to prevent seeds from dissolving in the mixture. A comprehensive study of seed stability and seeding techniques has been reported by Shaw Stewart *et al.* (2011 ▶).

Since the introduction of MMS there has been a debate about the mechanism by which crystal nucleation is promoted. It was proposed that modification of the crystallization conditions itself is the reason behind the increase in the number of hits since every condition in the screen is ‘biased’ towards the successful crystallization condition used to produce seeds (St John *et al.*, 2008 ▶). It was argued that using the original mother liquor without seeds could in principle give similar results.

While in some cases this is certainly true, there are other cases where the seeds themselves and not the mother liquor were the key factor to promote crystal growth. For instance, Fab 3-53/A27 was seeded by crystals of Fab 3-23/A27 and separately by those of Fab 3-53/B3. Both seed stocks contained 0.1 *M* MES pH 6.5, 18% PEG 3350 and 0.2 *M* NaAct/NaFmt; however, only Fab 3-23/A27 promoted crystallization, whereas Fab 3-53/B3 gave no hits in the same screens. Moreover, the hit was in 30% PEG 4000, 0.2 *M* AmSO4 with no buffer, indicating that neither of the components of the seed mother liquor could ‘bias’ nucleation.

Another example of ‘unbiased’ seeding is the case of Fab 5-51/O12. Various mixtures of seeds were tried to produce initial hits; however, only those where Fab 5-51/A27 was present, either alone or in combination with other proteins, succeeded. The effect of mother liquor can be ruled out because both components, ammonium sulfate and Tris buffer, were also present in mixtures that failed.

There are two possible positive outcomes of cross-seeding. In one, the nucleation phase is bypassed and crystal growth occurs directly on the seeds, resulting in crystals isomorphous to the seeds. This ‘isomorphous’ cross-seeding is exemplified by Fabs 3-53/O12, 5-51/L6 and 5-51/O12. Alternatively, either seeds or their mother liquor or both promote nucleation, which yields crystals non-isomorphous to the seeds. There were three such cases in our study: Fabs 3-53/A27, 3-53/L6 and 3-23/O12 (Fig. 2 ▶). Because all three cases involve a ‘common’ heavy or light chain, it was reasonable to expect that the ‘common’ chain defines key crystal contacts in both the seed and the target crystal forms. We examined all crystal contacts in these structures and found nothing in common with the corresponding ‘seed’ structure. While it is unclear how exactly MMS works, these examples demonstrate the power of MMS, as it may be successfully applied to stimulate hit formation even in non-isomorphous cases.

Based on our experience with MMS, we see the method as a significant step forward in macromolecular crystallization. MMS can increase the number of hits, minimize optimization time and in some cases improve crystal morphology. The method is highly recommended in both self-seeding and cross-seeding formats.

## Figures and Tables

**Table 1:** Crystal data

			Unit-cell parameters (Å)			
Fab	Resolution (Å)	Space group†	*a*	*b*	*c*	Crystallization conditions‡
1-69/B3	1.9	*P*42_1_2 (1)	120.1	120.1	64.2	HEPES pH 7.5, 10% PEG 8K, 8% ethylene glycol
1-69/A27	3.3	*P*422 (2)	152.5	152.5	123.4	MES pH 6.5, 2.1 *M* AmSO4, 5% MPD
1-69/L6	1.8	*C*2 (2)	212.1	55.1	80.3	MES pH 6.5, 25% PEG 3350, 0.2 *M* NaFmt
1-69/O12	2.6	*P*3_1_21 (1)	129.2	129.2	91.8	MES pH 6.5, 5.0 *M* NaFmt
3-23/B3	2.0	*P*2_1_2_1_2_1_ (2)	62.7	111.0	160.0	HEPES pH 7.5, 2.0 *M* AmSO4, 2% PEG 400
3-23/A27	2.2	*P*6_2_22 (1)	121.5	121.5	160.4	MES pH 6.5, 18% PEG 3350, 1.0 *M* LiCl
3-23/L6	2.0	*P*2_1_2_1_2_1_ (2)	60.9	110.6	158.8	NaAct pH 4.5, 2 *M* AmSO4, 5% PEG 400
3-23/O12	2.8	*P*4_1_2_1_2 (1)	96.6	96.6	105.4	20% PEG 3350, 0.2 *M* LiCtr
3-53/B3	2.5	*P*3_1_ (1)	68.1	68.1	95.6	MES pH 6.5, 16% PEG 3350, 0.2 *M* NaFmt, 5% MPD
3-53/A27	2.2	*P*6_5_22 (1)	89.4	89.4	211.7	NaAct pH 4.5, 19% PEG 4000, 0.2 *M* AmSO4
3-53/L6	2.3	*P*6_5_22 (1)	88.1	88.1	219.6	NaAct pH 4.5, 25% PEG 3350, 0.2 *M* Li_2_SO_4_
3-53/O12	2.7	*P*6_5_22 (1)	89.4	89.4	212.3	16% PEG 3350, 0.2 *M* AmSO4, 5% dioxane
5-51/B3	1.9	*P*2_1_ (2)	106.0	38.0	112.3	Tris pH 8.5, 24% PEG 3350, 0.2 *M* AmAct
5-51/A27	1.6	*P*2_1_2_1_2_1_ (1)	63.8	74.1	103.0	CHES pH 9.5, 1.0 *M* AmSO4
5-51/L6	2.5	*P*2_1_2_1_2_1_ (1)	64.3	73.8	103.0	Tris pH 8.5, 25% PEG 3350, 0.2 *M* MgCl_2_
5-51/O12	2.2	*P*2_1_2_1_2_1_ (1)	63.7	73.8	103.3	CHES pH 9.5, 1.8 *M* AmSO4, 5% dioxane

† The number of Fab molecules in the asymmetric unit is in parentheses.

‡ The buffer concentration is 0.1 *M* in all conditions.

## Footnotes

1 Abbreviations: AmAct, ammonium acetate; AmSO4, ammonium sulfate; AmTrt, ammonium tartrate; CHES, 2-(cyclohexylamino)ethanesulfonic acid; HEPES, *N*-(2-hydroxyethyl)piperazine-*N*′-2-ethanesulfonic acid; LiCtr, lithium citrate; MES, 2-(*N*-morpholino)ethanesulfonic acid; MPD, 2-methyl-2,4-pentanediol; NaAct, sodium acetate; NaCtr, sodium citrate; NaFmt, sodium formate; PEG, polyethylene glycol, XQ, X-ray quality.
